# A Morphometric Analysis of Starch Granules from Two *Dioscorea* Species

**DOI:** 10.3390/plants14121869

**Published:** 2025-06-18

**Authors:** Sara Rickett, Lisbeth A. Louderback, Adrian V. Bell

**Affiliations:** Department of Anthropology, Natural History Museum of Utah, University of Utah, Salt Lake City, UT 84108, USA; lisbeth.louderback@anthro.utah.edu (L.A.L.); adrian.bell@anthro.utah.edu (A.V.B.)

**Keywords:** archeobotany, *Dioscorea*, morphology, starch granules, yam, taxonomic identification, archeology, Tonga, Polynesia

## Abstract

*Dioscorea* is a genus comprising over 600 species, many of which possess edible tubers that are commonly referred to as yams. While *Dioscorea* is a significant crop across the globe, it holds a unique cultural significance to the people of Tonga in western Polynesia. Presently, *Dioscorea* is known for its essential role in festivals and ceremonies, as well as for its nutritional contributions to Tongan diets. To understand and to assess the significance of *Dioscorea* in the distant past, however, archeologists rely on plant residues (e.g., starch granules) preserved on ancient tools. This study provides the necessary first step in archeological starch analysis by examining the granule morphometrics of two culturally significant *Dioscorea* species, *D. alata* and *D. bulbifera* from Tonga. Tubers from three individuals of each species were collected on the island of Vava’u and processed for starch granule extraction and analysis. Morphometric characteristics, including two novel that describe shape (eccentricity ratio and hilum angle), were measured on approximately 300 granules per species. When statistically compared, these novel characteristics allow *D. alata* and *D. bulbifera* to be readily distinguished from one another, and therefore increase confidence in assigning archeological granules to a specific taxon.

## 1. Introduction

Throughout human history, starch-rich plant foods have been foundational to our diets. Plants with underground storage organs often provide a steady source of calories when cultivated or when gathered in the wild [1]. Determining the use of these resources in the archeological record, however, is challenging due to the ephemeral nature of herbaceous tissue. Starch analysis can provide insights by examining the microscopic granules of stored sugars left behind in soil, on artifacts, and even in dental calculus [2,3,4]. Starch granules can persist in soils and interstitial matrices in stone and shell tools for tens of thousands of years, even in warm, damp environments [2,3,5]. Careful examination of these granules can allow for taxonomic identification of the parent plant. The necessary groundwork for examining residues in archeological contexts is to quantitatively measure the morphological characteristics of reference starch granules from culturally significant plant species.

This study identifies and describes the starch granule characteristics of two species of yam (*Dioscorea alata* L. and *D. bulbifera* L.), a crop that has long been important to people living in remote Oceania and on the island nation of Tonga [6,7,8]. Although the archeology of this region is understudied, preliminary archaeobotanical analyses of soil [9,10] and shell scrapers have suggested the processing and use of tubers in the past [11]. Starch granule analysis also has the potential to illuminate which crops were preferred over time and could even provide insights into agricultural intensification and social stratification. The current study presents a systematic study of starch granules from two culturally significant yam species and provides the necessary groundwork to further explore the use of these food crops over time.

### Study Species

*Dioscorea* is a genus comprising over 600 species [12]. Several species possess edible tubers commonly referred to as yams. Although found throughout the tropics of Asia, North and South America, and Africa, it has been suggested that the origin of *Dioscorea* is in New Guinea based on the high diversity of species in the region [4,13]. *Dioscorea* remains an important crop in many places and maintains a unique cultural significance in the Polynesian country of Tonga [14].

**Figure 1:**
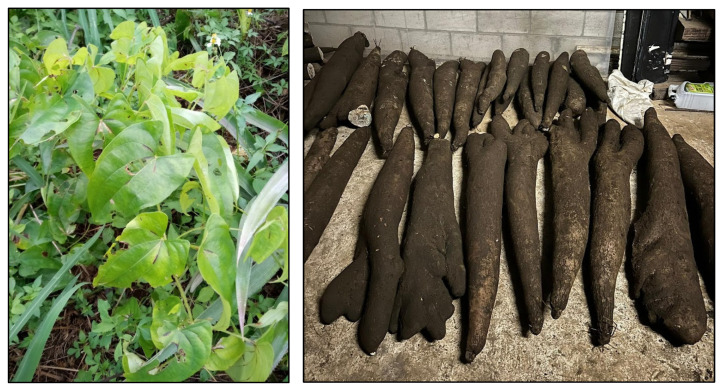
*D. alata* foliage in Tongan field (**left**). (Photo by S. Rickett). *D. alata* tubers harvested in June 2023 on Vava ‘u Island, Tonga (**right**). (Photo by Koliniasi Veamatahau).

**Figure 2:**
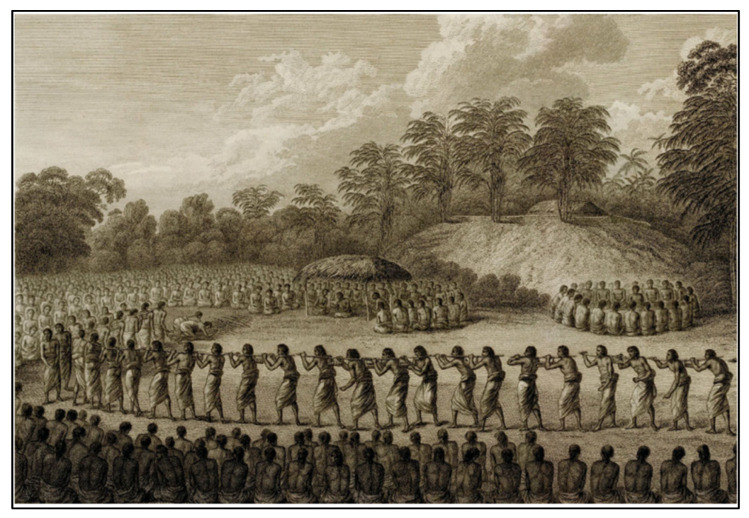
A depiction of the *‘inasi* ceremony held at Lapaha in 1777 by John Webber (Cook and King 1784) [18]. *D. alata* tubers are tied to poles carried by Tongan representatives.

The primary cultivated species in Tonga is *D. alata*, commonly known as the winged yam. *D. alata* is a highly esteemed and labor-intensive crop with tubers weighing up to 100 lbs. (Figure 1) [7,15]. This crop was noted during the first European contact accounts for its importance in annual festivals and its abundance. Accounts from William Mariner, an Englishman who lived in Tonga from 1806 to 1810, note the importance of this species in the *’inasi* or first fruits festival (Figure 2) [16]. This festival was central to Tongan political and religious life and was timed to coincide with the harvest of *D. alata*. The cultivation of *D. alata* includes lengthy preparation of arable fields since compact or rocky soil will inhibit the tuber’s growth. This indicates a level of agricultural security not indicated by less desirable crops such as *D. bulbifera* which has been identified as a famine food [10,17].

**Figure 3:**
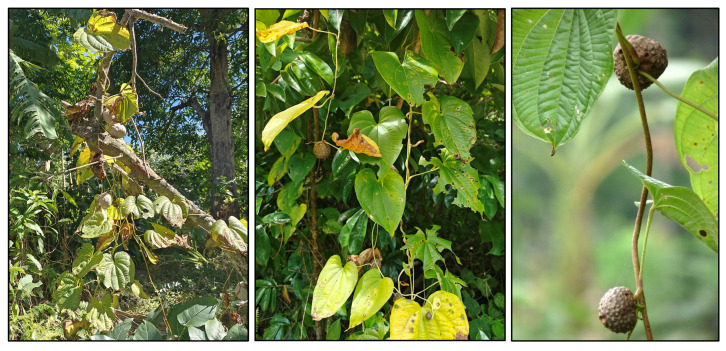
*D. bulbifera* with arial tubers growing in Vava’u (**left**) and Uiha (**center**), Tonga (photos by Sara Rickett). Close-up of aerial tubers (**right**) (Jee & Rani Nature Photography).

*D. bulbifera* is a weedy vine, frequently volunteering in fallow fields and uncultivated lands across Tonga (Figure 3). This species produces small aerial tubers that grow along the vine, dropping to the ground once they mature. It is noted for having toxic compounds that require special preparation to be edible. While no longer a commonly consumed crop, this persistent vine is part of the Polynesian assemblage of transported plants and was historically eaten in times of famine [17].

## 2. Materials and Methods

### 2.1. Reference Collection

Two species of *Dioscorea* that grow on the island nation of Tonga were selected for the present study. Plants (including their tubers) were collected in Tonga during May and June 2023 (Table 1). Home visits were made to local subsistence farmers, where interviews regarding agricultural practices were conducted. When possible, plantation visits were also conducted. The square footage of the growing plots was gathered via Garmin eTrex GPS, and rough tallies of crop species were collected. *D. alata* samples were collected from a local community garden in Vava’u and two additional household gardens. Although *D. bulbifera* is no longer an important food crop grown in Tonga, it was introduced to the islands by initial settlers and is a persistent volunteer. These specimens were collected from forested sections along roadsides.

### 2.2. Sample Preparation

A small portion (~1 cm^2^) of the tubers were pulverized with a clean glass mortar and pestle and deionized water (diH_2_O). Approximately 10 mL of diH_2_O was added to the sample, and the mixture was strained through a 125 µm U.S.A. standard test sieve to remove >125 µm debris. This mixture was then centrifuged for 3 min at 3000 RPM. The supernatant was decanted and discarded. The pellets were resuspended with 10 mL of diH_2_O using an analog vortex mixer. Samples were once again centrifuged at 3000 RPM for 3 min. The supernatant was decanted and discarded. Approximately 7 mL of a heavy liquid, lithium heteropolytungstate (LST) (Central Chemical Consulting, Malaga, Australia) with a specific gravity of 2.35, was added to each vial. The pellets were resuspended in the LST with a vortex mixer. The samples were then centrifuged for 20 min at 1000 RPM. This heavy liquid solution separates materials of varying densities, with lighter organics, such as starch granules, that float to the surface. Organic material was extracted (or collected) from the upper 1–2 mm of the vial and transferred to a new vial. This material was then rinsed with diH_2_O twice and centrifuged for 3 min at 3000 RPM, and the supernatant was decanted. A final rinse with acetone was performed to dry and sanitize the samples.

### 2.3. Microscopy

**Figure 4:**
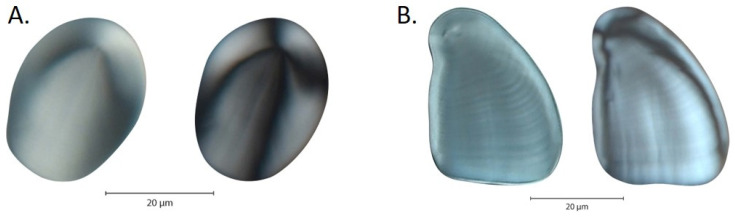
(**A**) *D. alata* granules in DIC (**left**) and polarized (**right**) light. (**B**) *D. bulbifera* granules in DIC (**left**) and polarized (**right**) light.

Starch granules were photographed with both polarized light and differential interference contrast (DIC) light (Figure 4). Polarized light highlights characteristics such as the hilum and extinction cross. Granule characteristics detected under DIC light include lamellae and fissures. Each slide was scanned using a transmitted brightfield microscope fitted with polarizing filters and Nomarski optics (ZeissAxioscope2, Zeiss International, Göttingen, Germany). A digital camera (Zeiss AxioCam HRc) with imaging and measurement software (Zen core v2.7) was used to capture images of, document, and measure the starch granules. Approximately 100 starch granules from each individual plant (n = 298 for *D. alata*, n = 301 for *D. bulbifera*) were measured and described. Granules were located via randomly generated microscope stage coordinates to avoid granule sorting due to fluid transport within the slide medium.

### 2.4. Granule Measurements and Morphology

Quantitative and qualitative characteristics were recorded for each starch granule, including size, shape, degree of eccentricity, and angle of hilum to width (Table 2; data available from Dryad). All quantitative measurements were made consistently by a single researcher using Zen core v2.7 software. The maximum length of each granule was measured through the hilum. The maximum width was measured at the widest part of the granule perpendicular to the maximum length.

**Figure 5:**
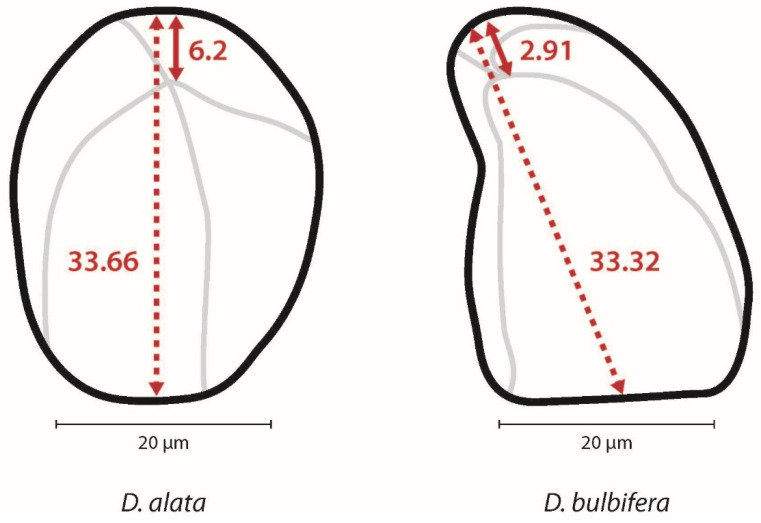
Eccentricity ratio (ER) is calculated by measuring the length from the hilum to the proximal end (solid line) divided by the maximum length through the hilum (dashed line). *Dioscorea alata* starch granule shows an ER of 0.18 and the *D. bulbifera* granule has an ER of 0.08.

**Figure 6:**
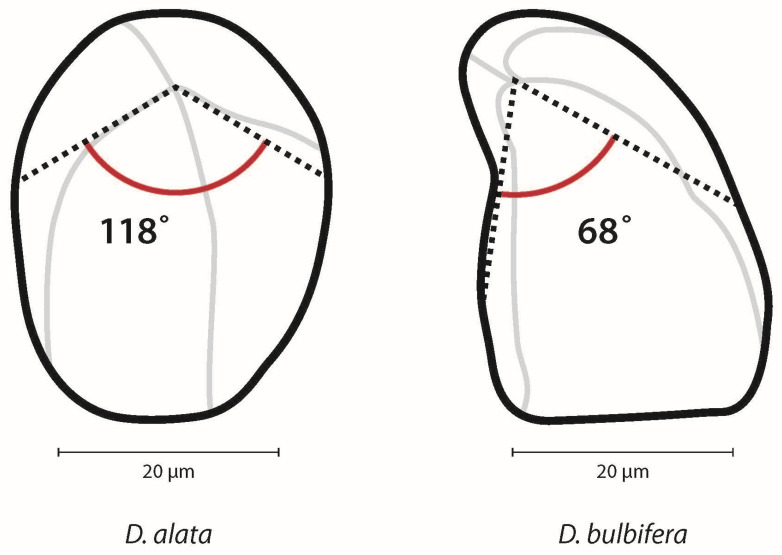
Angle of hilum is measured from the hilum to the widest part of the granule. *Dioscorea alata* granules tend to have a wider angle than *D. bulbifera* granules.

*Dioscorea* spp. starch granules almost exclusively possess an eccentric hilum (Table 2). The degree of eccentricity (eccentricity ratio) was calculated by measuring the length from the hilum to the proximal end (closest to the hilum) divided by the maximum length (Figure 5). The ratio ranges from 1.0, indicating that the hilum is far from the proximal end of the granule, to 0.0, indicating the hilum is very close to the proximal end. A strategy to further quantify of the differences in shape between *D. alata* and *D. bulbifera* is measuring the angle emanating from the hilum to the maximum width of the granule (angle of hilum) (Figure 6).

The frequency of qualitative morphological characters, including granule lamellae, longitudinal fissures, curves, and shape were recorded by a single researcher (Table 2). The terminology used follows that in Reichert 1913 and ICSN 2011 and is consistent with the published literature [10,19,20,21,22]. Lamellae are visible layers of amylose and amylopectin, which create concentric growth rings emanating from the hilum. Longitudinal fissures are anomalies in the granule progressing along the long axis of the granule, evident as lines of reduced light refraction along the granule. Curves, defined as smooth bends in form, frequently occur at the proximal end of the granule, near the hilum. Granule shapes include oval and triangular [21,22]. Oval granules display a convex curvature and are slightly more narrow at the distal end (farthest from the hilum). Triangular granules possessed three well-defined sides (Table 2).

Previous work [23] has demonstrated the utility of comparing starch granules from different species using only the longest 20% in terms of granule size (maximum length). This is because the abundance of small granules depends on the physiological condition of the storage organ (e.g., whether it is actively assimilating photosynthates and/or creating more plastids). Furthermore, large granules reflect genetic limits on granule size [24] and have more measurable and observable characteristics to distinguish between taxa. (Choosing a 20% subsample represents a compromise between improving character resolution and having an adequate number of granules—as a tool it could be further developed with studies on additional plant taxa). However, in the present study, we present data on the entire dataset of granule lengths (100% of sample) in addition to the 20% subsample. We tested for normality in all quantitative characters (length, width, eccentricity ratio, and hilum angle) and found a mix of normal and non-normal distributions (data available through Dryad and Zenodo). For example, in *D. alata*, none of these characters were normally distributed, but in *D. bulbifera*, granule length and width were near normal. Therefore, two-sample Kolmogorov–Smirnov (KS) tests were performed on the 100% sample and the longest granules in the 20% subsample when comparing size, eccentricity, and angle measurements, with *p*-values < 0.05 indicating significant differences.

**Table 2:** Qualitative morphological characteristics used in this analysis.

Morphological Character	Description	Image
**Hilum**	The hilum is the point of origin around which layers (lamellae) are deposited [22]. An eccentric hilum is located well outside the geometric center of the granule [21].	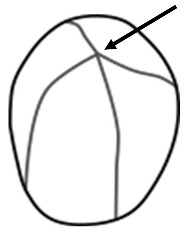
**Lamellae**	Concentric growth rings of amylase and amylopectin emanating from the hilum [21,22]. Visible under DIC light.	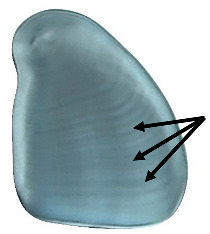
**Longitudinal Fissure**	Interruption in the starch granule, extending along the long axis of the granule [21,22].	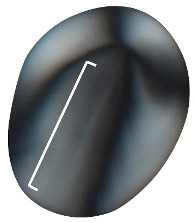
**Curve**	A curve or smooth bend in the granule, usually occurring on the proximal end of the granule close to the hilum.	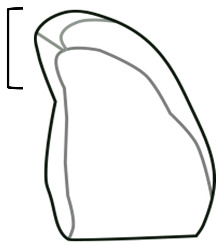
**Triangular**	Triangular granules possess three well-defined sides [21].	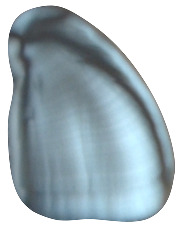
**Oval**	Oval granules have a rounded and slightly elongated outline [21].	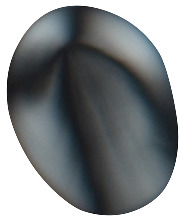

## 3. Results

### 3.1. Granule Characters and Morphology

The granules were analyzed based on quantitative metrics such as length, width, eccentricity ratio, and angle of hilum. Qualitative morphological characteristics including lamellae, longitudinal fissures, granule shape (oval or triangular), and granule curve were also recorded for every granule from each species. The frequencies of these characteristics in the entire dataset and the longest granules are shown in Table 3. The longest granules (20% subsample) possessed characteristics that were more frequently observed when compared to granules in the entire dataset (except for triangular shape in *D. alata* and oval shape in *D*. *bulbifera*). For the descriptions below, we report on the frequencies of characteristics possessed by the longest (20% subsample) granules.

### 3.2. Dioscorea alata

**Figure 7:**
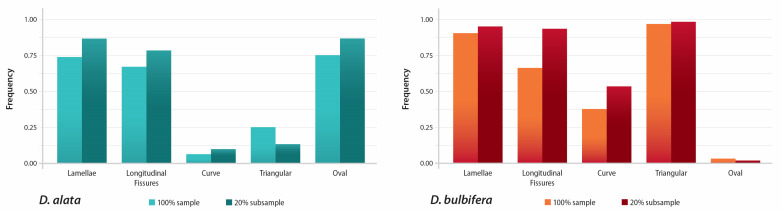
Frequency of morphological characters observed and documented in 100% and 20% subsamples of starch granules from *D. alata* and *D. bulbifera*.

**Figure 8:**
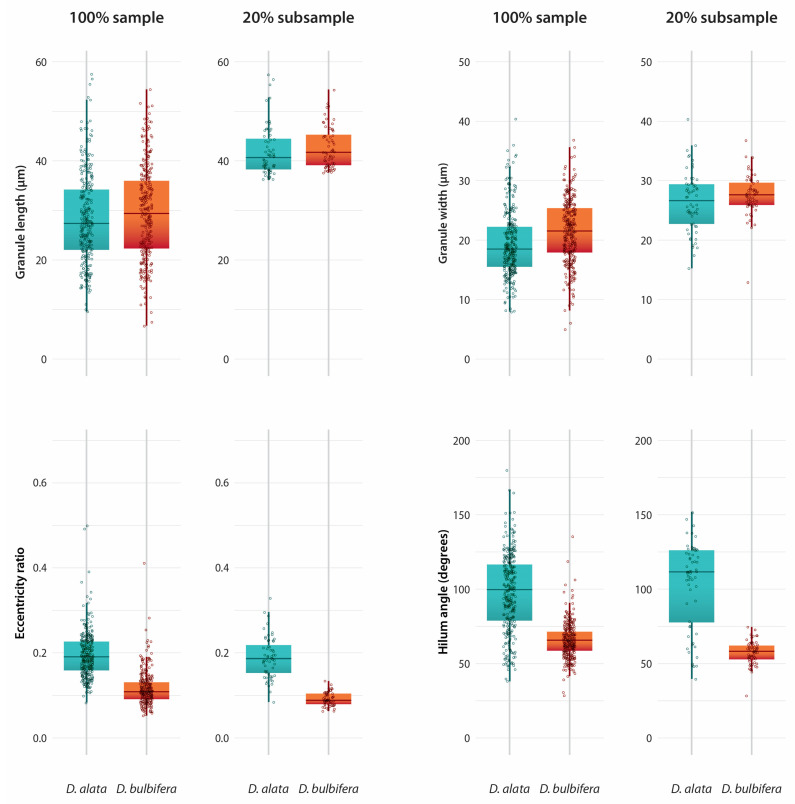
(**Top Row**): Size distributions for 100% of the sample and the 20% subsample of granule lengths and widths for *D. alata* and *D. bulbifera*. (**Bottom Row**): Distributions of the eccentricity ratio and angle of the hilum for 100% of the sample and the 20% subsample for *D. alata* and *D. bulbifera*. Data used for the box and whisker plot include median (center line in the box), the upper (75%) and lower (25%) quartiles (the box), the maximum and minimum values (whiskers), and all datapoints including outliers (points) [25].

Within the longest 20% of *D. alata* granules (n = 60), 86% were oval, while the remaining 14% were recorded as triangular. Longitudinal fissures were noted in 78% of *D. alata* granules. As few as 10% of granules were curved at the proximal end (Figure 7). The maximum length for the upper 20% sizes ranged from 36.35 to 57.46 µm (Figure 8). The mean eccentricity ratio was 0.19, with a range between 0.08 and 0.33. The mean angle from the hilum was 103°, with a range of 40–152° (Figure 8) (Table 3).

### 3.3. Dioscorea bulbifera

Dominant characteristics observed in *D. bulbifera* include oval to triangular granules, distinct lamellae, longitudinal fissures, and curved granules. Of the longest 20% of granules (n = 60), 98% were triangular. Longitudinal fissures were noted in 93%, and 53% of the granules were curved. Lamella were observed in 95% of granules (Figure 7). The maximum length of the 20% subsample of *D. bulbifera* granules ranged from 37.62 to 54.42 µm (Figure 8). The mean eccentricity ratio was 0.09, with a range of 0.06–0.13. The mean angle measurement was 58°, ranging from 28 to 75° (Figure 8) (Table 3).

### 3.4. Differences Between Species

The frequency of morphological characteristics such as hilum position and the presence of longitudinal fissures and lamellae do not significantly differ between taxa. However, *D. bulbifera* starch granules are more frequently curved and triangular when compared to *D. alata*, which do not often possess a curve and are more oval. The granule lengths in the 100% sample were marginally statistically significant with a K-S test *p* value of 0.046 and were not significantly different in the 20% subsample (K-S test, *p* = 0.265). The granule widths in the 20% subsample are significantly different between taxa (K-S test, *p* = 0.008) (Figure 8). The distributions of granule lengths and widths overlap for both taxa regardless of whether 100% of the sample or the 20% subsample are examined, which complicates the use of these metrics as potential diagnostic characteristics. The eccentricity ratio and hilum angle are significantly different between *D. alata* and *D. bulbifera* regardless of the sample size (100% vs. 20%) (K-S test, *p* < 0.0001 for all comparisons) (Figure 8). However, when the 20% subsample is analyzed, there is significantly less overlap in the distribution of measurements. For example, the eccentricity ratio is significantly higher in *D. alata* (0.08–0.33), indicating that the hilum is more centrally located within the granule in comparison to *D. bulbifera* (0.06–0.13), where the hilum is closer to the proximal end (K-S test, *p* < 0.0001) (Figure 8). Likewise, the hilum angle for the 20% subsample ranges from 40° to 152° in *D. alata* compared to 28° to 75° in *D. bulbifera*. These two measurements introduce novel characters that highlight the distinct morphologies of the two species, thereby increasing confidence in taxonomic assignments.

## 4. Discussion

This study examines the morphometrics of starch granules from two species of *Dioscorea*, *D. alata* and *D. bulbifera*. The analysis highlights the value of quantifying characteristics beyond classic descriptors such as length and width of granules. While these metrics have been a standard used to describe and assign taxonomic identity to ancient starch granules [2,19,26], our understanding of the complexity and nuance of granule morphology is still developing. The metabolic dynamics that affect growth and maturation (ultimately leading to maximum size and characteristic expression) remain obscure, as do the mechanisms of genetic control as layers of carbohydrate accrete around the hilum. Populations of granules are thus heteromorphic and necessitate subsampling to examine species-specific features on those which are fully formed (20% subsample). Conducting systematic studies on reference granules from culturally significant plant taxa increases our confidence when making taxonomic assignments for archeological granules. In this case, calculating and statistically comparing the eccentricity ratio and the hilum angle in combination with the qualitative characters, allows *D. alata* and *D. bulbifera* granules to be readily distinguished from one another.

Previous analyses of *D. alata* and *D. bulbifera* starch granules reveal similar results for granule size measurements, but differ from the present study in terms of morphological descriptions of the granules and documentation of characteristics [10,19,20,27]. For example, Ussher analyzed several *Dioscorea* species, including *D. alata* and *D. bulbifera*, as well as many other traditional Polynesian crops [10]. That study reported size ranges consistent with those found in this analysis; however, our measurements are slightly larger. Ussher also calculated an eccentricity ratio for both species. Those calculations are comparable to the present study, except in the case of *D. alata* starch, which showed a higher degree of eccentricity. Ussher describes *D. alata* granules as predominantly lenticular and *D. bulbifera* granules as lenticular or wedge-shaped. Notable characteristics documented in the study consist of stellate fissures, faceting, equatorial grooves, and lamellae. A measurement defined as the “maximum angle within the arms of the extinction cross” [10] (p. 83) was mentioned, and may be analogous to the hilum angle used in this paper, but ultimately was not used as a metric variable. Field and colleagues [28], explore several different metrics (size, shape, morphological characters) of starch granules from a reference collection in Papua New Guinea, including *D. alata* and *D. bulbifera.* The size (maximum length) of granules from both species were comparable to the current study; however, the maximum length in *D. alata* was greater. Morphological characteristics were also analyzed in Field et al.’s study, and similar to our findings, *D. bulbifera* granules possess a more eccentric hilum when compared to *D. alata* [28].

Another study (Mercader and colleagues [19] described starch granules from numerous taxa including *D. alata* and *D. bulbifera* using novel 3D and 2D morphotypes. Granules from both taxa predominantly exhibited the same morphotype, Parabolic Prism (3D) and Parabolic (2D). *D. alata* starch had more variation in morphotype than *D. bulbifera*, with granules fitting into five morphotypes compared to three for *D. bulbifera*. Parabolic morphotypes appear to be exclusive to Dioscoreaceae, leading to potential use for family-level taxonomic identification. The granule sizes reported by Mercader and colleagues were similar to the present study, but again, our maximum lengths and widths were greater [19]. Fatokun explored utilizing amylose and amylopectin ratios within starch granules as a way to assign taxonomic levels with some promise in differentiating *D. alata* and *D. bulbifera* [20]. However, research in this technique is limited and its use in ancient starch is largely unexplored. Size distributions for *Dioscorea* starch granules reported in Fatokun’s study were similar to the ones reported in the present study [20]. No additional measurements on the granules were noted and no morphological characteristics were discussed in depth. Overall, these datasets show consistent granule sizes for *Dioscorea*, but highlight inconsistencies in shape descriptions and the recording of morphological characters. This is not surprising due to the qualitative nature of such data. The present study suggests that size and presence-absence of morphological characters alone are not sufficient for species-level identification. Qualitative shape designations may be effective if applied uniformly; however, the few studies conducted on *Dioscorea* starch over the past decade have not used consistent nomenclature or methodology. This leads to significant barriers to replicating research and statistically comparing results.

When employed in artifact residue analysis, determining taxa can elucidate aspects of past social ecology, artifact interpretation, agricultural intensification, and dietary analysis. Future work will focus on expanding this study to include a larger assemblage of Polynesian crops to help answer broader questions about Tongan prehistory. The two crops selected for this study are nurtured in highly different contexts, with *D. alata* generally flourishing in complex, stratified societies [29], and *D. bulbifera* serving as a more expedient, utilitarian crop [30]. Reliable and replicable determination methods such as the ones explored in this paper will promote accuracy in archeological starch research and lay groundwork for future study.

## Figures and Tables

**Table 1:** Sample number, *Dioscorea* species, Tongan name, and collection location (island) for reference material analyzed in the current study.

Lab Sample Number	Species	Variety/Tongan Name	Island	Context
LS1	*D. alata*	*‘Ufi Sikalu*	Vava’u	Community Garden
LS2	*D. alata*	*‘Ufi Hawaii* (purple var.)	Vava’u	Private plantation
LS3	*D. alata*	*‘Ufi Hawaii* (white var.)	Vava’u	Private plantation
LS5	*D. bulbifera*	*Hoi*	Vava’u	Roadside, fallow field
LS6	*D. bulbifera*	*Hoi*	Vava’u	Fallow field
LS7	*D. bulbifera*	*Hoi*	Vava’u	Roadside

**Table 3:** Starch granule measurements (granule length, eccentricity ratio, and angle of hilum) and frequency (%) of morphological characters for 100% and 20% subsample of granules from each taxon.

Plant Taxon	Granule Length	Eccentricity Ratio (Range)	Angle (Range)	Shape	Lamellae	Longitudinal Fissure	Curved at Proximal End
*D. alata* (100%) (n = 298)	9.53–57.46 µm	0.08–0.50	38–180°	Oval 75%	73%	67%	6%
*D. alata* (20% subsample) (n = 60)	36.35–57.46 µm	0.08–0.33	40–152°	Oval 86%	86%	78%	10%
*D. bulbifera* (100%) (n = 301)	6.65–54.42 µm	0.05–0.41	28–135°	Triangular 96%	90%	93%	37%
*D. bulbifera* (20% subsample) (n = 60)	37.62–54.42 µm	0.06–0.13	28–75°	Triangular 98%	95%	98%	53%

## Data Availability

The original data presented in the study are openly available in Dryad at https://doi.org/10.5061/dryad.n8pk0p351 and in Zenodo at https://zenodo.org/records/14171046.
